# Addressing Care Continuity and Quality Challenges in the Management of Hypertension: Case Study of the Private Health Care Sector in Kenya

**DOI:** 10.2196/18899

**Published:** 2021-02-17

**Authors:** Aisha Walcott-Bryant, William Ogallo, Sekou L Remy, Katherine Tryon, Winnie Shena, Marloes Bosker-Kibacha

**Affiliations:** 1 IBM Research Africa Nairobi Kenya; 2 CarePay Limited Nairobi Kenya; 3 Kenya Obstetrical and Gynecological Society Nairobi Kenya; 4 Africa Health Business Nairobi Kenya

**Keywords:** hypertension, health information systems, mobile phone, private sector, Kenya

## Abstract

**Background:**

Hypertension is a major risk factor of cardiovascular disease and a leading cause of morbidity and mortality globally. In Kenya, the rise of hypertension strains an already stretched health care system that has traditionally focused on the management of infectious diseases. Health care provision in this country remains fragmented, and little is known about the role of health information technology in care coordination. Furthermore, there is a dearth of literature on the experiences, challenges, and solutions for improving the management of hypertension and other noncommunicable diseases in the Kenyan private health care sector.

**Objective:**

The aim of this study is to assess stakeholders’ perspectives on the challenges associated with the management of hypertension in the Kenyan private health care sector and to derive recommendations for the design and functionality of a digital health solution for addressing the care continuity and quality challenges in the management of hypertension.

**Methods:**

We conducted a qualitative case study. We collected data using in-depth interviews with 18 care providers and 8 business leads, and direct observations at 18 private health care institutions in Nairobi, Kenya. We analyzed the data thematically to identify the key challenges and recommendations for technology-enabled solutions to support the management of hypertension in the Kenyan private health sector. We subsequently used the generated insights to derive and describe the design and range of functions of a digital health wallet platform for enabling care quality and continuity.

**Results:**

The management of hypertension in the Kenyan private health care sector is characterized by challenges such as high cost of care, limited health care literacy, lack of self-management support, ineffective referral systems, inadequate care provider training, and inadequate regulation. Care providers lack the tools needed to understand their patients’ care histories and effectively coordinate efforts to deliver high-quality hypertension care. The proposed digital health platform was designed to support hypertension care coordination and continuity through clinical workflow orchestration, decision support, and patient-mediated data sharing with privacy preservation, auditability, and trust enabled by blockchain technology.

**Conclusions:**

The Kenyan private health care sector faces key challenges that require significant policy, organizational, and infrastructural changes to ensure care quality and continuity in the management of hypertension. Digital health data interoperability solutions are needed to improve hypertension care coordination in the sector. Additional studies should investigate how patients can control the sharing of their data while ensuring that care providers have a holistic view of the patient during any encounter.

## Introduction

### Background

Being the leading cause of morbidity and mortality globally, noncommunicable diseases (NCDs) are a significant global health concern in sub-Saharan Africa and other parts of the world. It is projected that by 2030, deaths from NCDs in sub-Saharan Africa will exceed the combined deaths of communicable and nutritional diseases and maternal and perinatal deaths [1]. The United Nations’ sustainable development goal target 3.4 requires that by 2030, all member countries should have reduced premature mortality from NCDs by one-third (baseline 2015) through prevention and treatment and should promote mental health and well-being [2]. However, a 2020 study showed that in most countries, the progress toward achieving this target is slow and that getting back on track requires a combination of prevention, early detection, and treatment within accessible and equitable health systems [3].

This study focuses on hypertension, a major risk factor of cardiovascular disease and one of the most important contributors to NCD-related deaths [4]. By 2015, an estimated 1.13 billion people had hypertension globally [5]. Africa has an estimated hypertension prevalence of 30% among people 15 years or older [6]. This region disproportionately bears the largest burden of the condition, and it is projected that by 2030, over 216 million people in sub-Saharan Africa will have hypertension [7]. In Kenya, the age-standardized prevalence of hypertension is estimated to be 24.5% [8]; however, only 15% are aware of their status and only 8% are on treatment [9]. As there is no cure for hypertension, patients require life-long, continuous care, including screening, access to medication, regular testing, and personalized lifestyle plans.

In response to the impending cardiovascular disease crisis in Kenya, the Kenyan government has developed care guidelines to standardize the management of cardiovascular disease and committed to supporting public and private sector stakeholders to ensure implementation of the guidelines [10]. However, a review of the Kenyan policies on cardiovascular diseases revealed key gaps such as lack of integration with primary health care, lack of private sector involvement, and lack of integrated systems for patient referrals and health data sharing [11]. Furthermore, the Kenyan Ministry of Health identified some critical issues with the current health system in addressing the growing burden of NCDs [12]. These include (1) poor prioritization of NCDs throughout the health care system; (2) poor availability and affordability of safe and efficacious basic technologies and medicines for screening, diagnosis, treatment, and monitoring of NCDs; and (3) inadequate capacity of the health workforce in terms of numbers, equipment, and skills mix for the prevention and control of NCDs.

### Objectives

Few studies have investigated the management of hypertension in the Kenyan private health sector in Kenya, despite the sector’s fast growth driven by increasing demand for quality health care. It is estimated that up to 42% of the health workers provide services in private facilities [13] and that up to 47% of the poorest Kenyans are seeking care from private facilities [14]. However, there is a dearth of literature on the management of NCDs and associated risk factors in the private health sector of Kenya. Little is known about the experiences of care providers in this sector and the challenges they face and how health information systems can be used to address these challenges.

We carried out a case study that aims to understand the management of hypertension in the private health sector in Nairobi, Kenya. The objectives of this study are two-fold. The first objective is to identify and characterize the challenges in the management of hypertension as perceived by health care professionals in the private health sector. The second objective is to derive recommendations for the design and functionality of a digital health information exchange and decision support platform for addressing care continuity and quality challenges in the management of hypertension and other chronic illnesses. This paper reports our findings.

## Methods

### Study Design

This descriptive case study used a multimethod approach involving in-depth stakeholder interviews and direct observations. This study was conducted at 18 health care institutions located within the greater Nairobi area of Kenya and providing care to patients of low-, middle-, and high-socioeconomic status. These included 6 pharmacies, 6 general health clinics, and 6 specialist outpatient clinics associated with hospitals. The chosen institutions were purposefully selected by the study team such that each socioeconomic stratum was represented by 2 pharmacies, 2 general clinics, and 2 specialist clinics. The study was designed by a team consisting of domain experts from different disciplines, including health care, user interaction and design, and computer science. To mitigate bias, questions were mainly focused on the industry and not specific institutions, and all responses were anonymized.

### Data Collection

Data were collected by 5 nurses who were recruited through the National Nurses Association of Kenya and trained by the study team. Of the 5 nurses, 3 collected data from 4 facilities, and 2 collected data from 3 facilities, giving a total of 18 facilities. All the institutional visits were carried out for the purpose of research data collection only, and none of the 5 data collectors had affiliations with any of the facilities studied.

In each location, 4 key processes were carried out for this qualitative study:

1. In-depth interviews: an in-depth interview was conducted with the lead health care professional at the location to understand their education, training, and experience. In addition, the interviews aimed to understand the health care professional’s management of patients with hypertension, tools available to them in their workplace, and the challenges they face in managing patients with hypertension.
2. Roleplay: a roleplay exercise was conducted with the lead health care professional to observe their routine management and care of (1) a patient newly diagnosed with hypertension and (2) patients with hypertension requiring a routine.
3. Facility tour: a facility tour was conducted to identify and understand what resources were available within the facility to serve patients with hypertension.
4. Contextual observations: observations were recorded about the location to understand the context in which the health professionals work and where patients visit.

In addition, interviews were conducted with the business leads of 8 institutions to understand their perspectives on the increasing prevalence of NCDs and to understand the management through their institution. For each location, detailed notes were taken against the interview questions, role-play guides, tour guides, and observation guides.

### Data Analysis

All research notes were collated and manually analyzed using the thematic analysis guide by Braun and Clarke [15]. The goal of the analysis was to discover themes about the challenges and gaps associated with the management of hypertension at the study sites. Specifically, the analysis aimed at exploring participants’ perspectives on how they managed suspected and confirmed cases of hypertension and why this process was challenging. Clusters of semantically similar features and responses were first identified from the transcribed research notes. Next, the specific potential themes and subthemes were identified from the clusters. Subsequently, the potential themes and subthemes were iteratively verified against the original data. Finally, the final themes and subthemes were defined, refined, and reported. These themes and subthemes were used to inform the design and development of a novel platform to support the management of NCDs, provide continuity of care across facilities and systems, and improve the quality of care.

## Results

### Hypertension Management Challenges in the Kenyan Private Health Care Sector

Through this study, we identified 6 themes and 17 subthemes regarding the challenges in the management of hypertension in the private health care sector in Kenya. These are summarized in Table 1 and discussed below.

**Table 1:** Themes and subthemes about the management of hypertension in the private health care sector in Kenya.

Theme	Subtheme
High cost of hypertension management	Limited resources introducing priorities that compete with hypertension management High cost of medications prevents effective management of hypertension Prescribing habits and patient preferences fail to mitigate the cost of medications
Limited health literacy	Ignorance about hypertension prevents effective management of the disease Need to address health literacy and improve hypertension awareness
Lack of self-management support	Patients lack access to their hypertension management data Patients lack access to postappointment information for self-management Lack of tailoring of advice to patients due to limited capacity to monitor patients in the community Limited off-site follow-up and outreach of patients in the community
Ineffective referral systems	Patients perceive self-referral to higher-level hospitals as acceptable Knowledge and skill gaps exist at the different levels of the health care pyramid There is no financial incentive for private hospitals to refer patients back to the community or lower levels of care Litigation fears around task shifting hinder downward referrals to community hospitals
Inadequate care provider training	Lack of adequate training on the management of hypertension Variability in decision-making aids
Inadequate regulation	Lack of adequate regulations on the quality of services in private health facilities High number of unregulated pharmacies

#### High Cost of Hypertension Management

We found that the cost of a hypertension consultation at a private clinic or hospital in the greater Nairobi area ranges from US $4 to US $40. Patients are encouraged to attend a monthly consultation for progress monitoring and renewal of prescriptions. Most payments are out of pocket; thus, the cost of care affects the frequency of consultations. Some respondents stated that consultation fees place a significant burden on patients with lower income levels. Often, health care budget burdens compete with other priorities such as housing and education (subtheme 1):

Health is just not a priority for our clients, they [patients] are worrying about education and housing, not health. They come in when they absolutely have to.

Healthcare Group General Manager

It is expensive for people to keep going back to the hospital, so they stop going and either stop their medication or keep buying it without a checkup.

Pharmacy CEO

An additional financial issue in the management of hypertension is the cost of medication (subtheme 2). Seven of the 18 health care professionals interviewed stated that medication cost was one of the biggest factors preventing their patients from effectively managing their hypertension. In addition, some respondents noted that habits such as prescribing branded drugs fail to mitigate the costs of medications (subtheme 3):

Doctors write branded prescriptions and patients insist on receiving medication that is written on the prescription, even if there is a cheaper generic version available.

Pharmacy CEO

#### Limited Health Literacy

Nine of the 18 health care professionals interviewed identified limited knowledge about hypertension and its management as the primary factor preventing their patients from effective management of their condition (subtheme 4):

Ignorance in the market is the biggest issue in managing chronic disease.

Clinic General Manager

No one understands blood pressure, what it means and how to manage it.

Pharmacy CEO

If you try and screen patients, they think you are over investigating them for more money.

Clinic General Manager

Respondents recognized the need to address the health care literacy of their patients to improve hypertension management (subtheme 5). For example, most interviewed pharmacists identified their role as critical for patient education and follow-up. They also identified specific challenges within their role in patient education. These included (1) limited access to patient health history, (2) lack of education and training in the management of NCDs, and (3) potential conflicts of interest in selling medications. Other approaches for improving health literacy included learning from experiences in the management of other chronic illnesses such as HIV/AIDS and educating patients during routine care visits:

We need to learn from the HIV work on how to get the prevention message out.

Hospital CEO

We offer health talks in companies for our capitated patients as it is worth our while doing that.

Healthcare Group General Manager

#### Lack of Self-Management Support

This study identified several gaps pertaining to tools for supporting patients with hypertension. First, patients lack easy access to their hypertension management health records (subtheme 6). During the interviews, the health care professionals acknowledged that some patients used apps and notebooks to record blood pressure measurements. Most patients can obtain printed copies of their care notes on request from the institution. However, one institution forbade access to health records by patients:

Records are given [to patients] by word of mouth and files not issued out due to privacy purposes.

Hospital CEO

Second, the health care professionals interviewed identified the incompatibility of health records between institutions as another issue. Specifically, the respondents noted that the transfer of records between institutions is a complicated process. As a result, important details of patient cases can be missed and investigations can be repeated, leading to additional medical costs for patients and their insurers.

Third, patients lack information for supporting self-management once they leave the doctor's office (subtheme 7). Health care professionals counseled their patients on lifestyle and medication adherence at meetings or appointments. However, there were very few take-home materials for their patients. Of the 18 institutions interviewed, only 2 had leaflets or pamphlets on hypertension. The fourth gap is on counseling, where generic advice on lifestyle changes and medication adherence was given rather than personalized advice (subtheme 8). Interviewees noted that they see a limited view of their patients' lives, and there is little monitoring of patients in the community.

The final gap identified was limited to follow-up and outreach with patients in the community (subtheme 9). One hospital had phone call outreach and counseling, one clinic had a monthly health education session for patients with hypertension, and one health care group had health talks for their capitated groups. In total, 15 out of 18 institutions relied entirely on appointments to educate and assist patients with the management of their condition:

There is no active outreach into the community; the hospital waits for patients to come back for an appointment.

Hospital CEO

Many patients fall through the cracks and only come back with serious consequences of their condition.

Hospital CEO

#### Ineffective Referral Systems

The Kenyan health care system is organized in a *pyramid of care*, where less complex cases should be managed in a community or primary care setting and increasingly complex cases should be referred to general or specialist hospitals. We found that more complex cases, with greater levels of comorbidities and complications, were seen in hospitals rather than clinics. However, the interviewees acknowledged that many uncomplicated or well-controlled cases are also managed in hospitals, thereby resulting in higher costs and inefficient use of resources. The reasons why many patients receive care in hospitals, rather than in clinics, are discussed below. First, many patients perceive self-referrals to higher-level hospitals as acceptable (subtheme 10). We found that patients bypass lower-level facilities because they think that they would be referred to a higher-level facility and that it is easier and more cost-effective to go straight to higher-level institutions:

Patients realize that lower-cost options often result in referrals costing more in health, time and money, so they prefer to come straight to hospital.

Hospital CEO

It is not acceptable in the market to see a clinical officer or a nurse manage your condition, people expect to see a medical officer.

Healthcare Group CEO

Patients think that more expensive is better health care and we do not change that perception, so they keep coming back to us for routine care.

Hospital CEO

It is about giving patients what they want.

Healthcare Group CEO

Second, there are skill gaps in the health care workforce at different levels of the care pyramid. For example, respondents stated that there are care providers at lower-level hospitals who do not have the skills necessary for managing hypertension. Other respondents identified the lack of a specialized skilled health workforce at higher-level hospitals as a significant barrier to the management of hypertension:

There is no truly skilled primary care in the community to manage NCDs.

Hospital CEO

Community health workers screen in the community, but patients have to come to the clinic for follow up.

Clinic General Manager

We have a significant shortage of skilled staff at the subspecialty level in chronic disease.

Healthcare Group General Manager

Third, it was broadly acknowledged by interviewees that there is no financial incentive for private hospitals to refer patients with hypertension back to the community- or lower-level facilities, even if they are well managed (subtheme 12). Hospitals themselves have little incentive to task shift within their institutions and to use lower cadres of professionals in the health care system as insurers to date are not generally willing to accept capitated services and few providers offer capitated services to their customers. Capitation refers to a payment contract between an insurer and a care service provider in which the insurer periodically pays the service provider a fixed amount for an enrolled person regardless of whether or not the patient seeks care. Respondents opined that the lack of capitation discourages task shifting, possibly because of the associated hospital revenue losses:

Insurers are not yet putting pressure on us to reduce the cost of care.

Hospital CEO

Insurers have not historically accepted capitated rates for their customers in return for a lock-in to [named health care group] services, so we have no incentive to task shift.

Healthcare Group CEO

Fourth, the fear of litigation around task shifting hinders the referral of patients seen at higher-level facilities back to the community (subtheme 13). Such fears are associated with the lack of policies governing referrals from higher-level hospitals back to lower-level facilities:

There is some pressure from NHIF and insurers to reduce the cost of care, but we still have doctor-led service due to litigation worries about task shifting to lower cadres of professionals.

Hospital CEO

We would wait for policy change at the government level before risking task shifting.

Hospital CEO

#### Inadequate Care Provider Training

This study highlighted specific concerns regarding inadequate training on NCDs and lack of continued professional development (CPD; subtheme 14). For example, 8 of the 18 health care professionals interviewed took no additional training in hypertension management after their initial qualification. Of the remaining 19 who had some training, 4 received training via CPD organized by pharmaceutical companies and focused on companies’ branded medications. This situation was even more severe for pharmacy staff interviewed, with 4 of 6 staff having no further training following qualification:

Most CPD is provided by pharmaceutical companies trying to promote their medication, and there is a critical need for independent CPD provision.

Hospital CEO

CPD should be regulated by separate boards from those providing the CPD. Currently, many of the professional bodies both provide and regulate CPD.

Hospital CEO

CPD is not obligatory for healthcare professionals. In the case of re-licensing, most [professionals] qualify by completing their required hours. So many do not continue to learn after they have completed their initial training. This is a particular issue for NCDs as little of their initial training would have covered these at a time when HIV, TB, and Malaria were the main focus.

Hospital CEO

Our research also identified a large variability in the decision-making aids used by different health care professionals to guide the care of their patients (subtheme 15). For example, of the 18 institutions interviewed, each used different sets of guidelines to manage their patients with hypertension. This highlighted not only the inconsistency in training across the board but also the challenges faced by patients moving between institutions that follow different guidelines.

#### Inadequate Regulation

During the interviews, there were important regulatory concerns that were identified. First, although health care professionals in private facilities are regulated by their respective professional bodies, the facilities themselves are not regulated for quality of service (subtheme 16):

There is no quality inspection of private facilities, so as long as you pass financial audit you can stay open regardless of the quality of care. An independent quality commission is much needed.

Hospital CEO

We worry about bad newspaper articles that will stop people coming to our hospital, not a quality audit. We need to keep our patients happy.

Hospital CEO

Second, the Pharmaceuticals and Poisons Board has been tasked with the regulation of the pharmacy industry in Kenya. However, there remain significant issues and a large number of unregulated pharmacies across the country (subtheme 17):

Some wholesalers insist on seeing licensing before they sell pharmaceuticals, but others do not, so you can still sell pharmaceuticals without a license.

Pharmacy CEO

The Pharmacy and Poisons Board announce their investigations the week before they do them so many pharmacies are closed when they come to investigate.

Pharmacy CEO

Patients do not know who is legitimate and who is not. No one explained what the green cross system meant.

Pharmacy CEO

### Proposed Approach to Addressing Care Continuity and Quality Challenges

A total of 22 recommendations were identified from the solutions described by the interviewees in this study (Table 2). Of these solutions, 6 directly involved the use of health information systems to address different aspects of care: (1) reducing costs of hypertension management (recommendation 3), (2) supporting continuous patient follow-up and education (recommendation 10), (3) enabling self-measurements (recommendation 15), (4) enhancing patient adherence (recommendation 16), (5) supporting interoperability for health facilities and systems to support the health care pyramid (recommendation 17), and (6) adoption of local clinical guidelines (recommendation 18).

**Table 2:** Solutions for improving management of hypertension recommended by interviewees.

Theme	Recommendations
High cost of hypertension management	More affordable or subsidized medications Greater penetration of insurance coverage Technology solutions to reduce the cost of care, for example, telemedicine, self-service machines, monitoring and triaging patients, and advising patients (eg, on generic medications) Financial incentives for technology use over in-person visits for health care professionals or organizations Incentive schemes for individuals to make lifestyle changes and be compliant with medications Improved infrastructure so that people can walk more frequently and more safely National policies around healthy food Improved financial access to healthy food
Limited health literacy	Use of nurse educators Technology to support continuous patient follow-up and counseling Education materials, including videos in the waiting bays in clinics or hospitals, and materials for the general public Increasing the role of pharmacists in patient education Educating children in school about health and healthy lifestyle
Lack of self-management support	Introduction of patient held records Kiosks for patients to measure themselves independently Tracking devices for patient lifestyle and medication adherence monitoring in the community
Ineffective referral systems	Vertical integration between different tiers of care delivery through efforts such as enabling communication and cooperation between care providers, improving referral care coordination and supervision, and providing transportation
Inadequate care provider training	Adoption of local guidelines Training of pharmacy staff on the management of NCDs^a^ Independent CPD^b^ for health care professionals Compulsory CPD linked to licensing
Inadequate regulation	Greater regulation of health care organizations (including pharmacies) on quality

^a^NCD: noncommunicable disease.

^b^CPD: continued professional development.

The themes and recommendations identified by our research suggest that the inherent complexities of Kenya’s health care landscape make it difficult for stakeholders to ascertain a deep understanding of the patient’s condition and effectively coordinate efforts to deliver high-quality care. This reveals the overarching lack of capacity for enabling care quality and continuity within a fragmented ecosystem. In this regard, we identified the need to strengthen health systems in the Kenyan private and public sectors to support the management of hypertension and to ensure care continuity and quality within and across facilities. On the basis of the lessons learned from our research, we proposed a patient-centered platform, called the *Digital Health Wallet Platform*, which aims to improve the documentation and exchange of health data gathered during care provision by allowing patient-consented and mediated data sharing [16]. The platform is built on top of a blockchain network to ensure privacy and auditability of care provision.

This platform has 3 core components: a care management platform, a patient care wallet, and a clinical encounters app (Table 3). The *care management platform* manages clinical encounters and enables the patient-controlled exchange of health data via blockchain protocols while adhering to the Fast Healthcare Interoperability Resources (FHIR) version 3.0 standard [16]. It supports data sharing and guideline-based decision making in outpatient clinical workflows. The *patient care wallet* is a mobile app where patients view their health records and can track who accessed their records [16]. It also enables patient-consented data sharing via a decentralized consent management blockchain system. Finally, the *clinical encounters app* supports care providers through workflows and enables requests for patient consent to share health data between providers [16].

**Figure 1:**
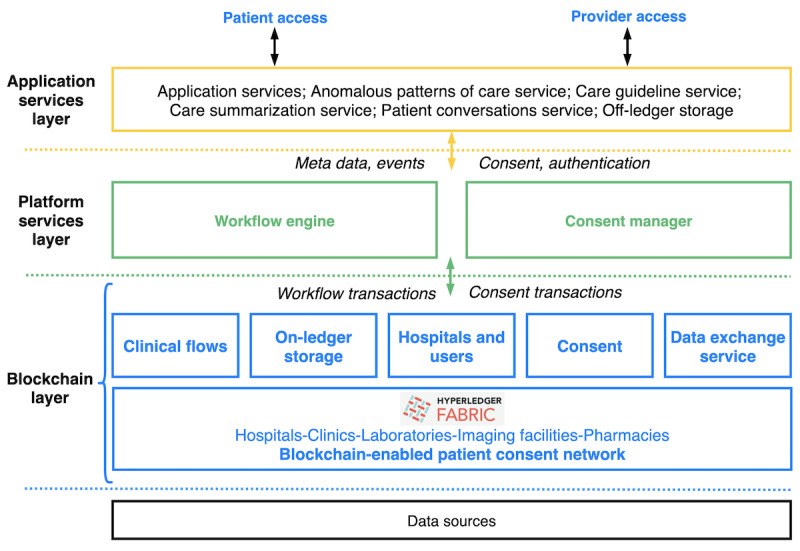
High-level overview of the proposed digital health wallet platform for enabling care continuity.

The architecture of the proposed platform is illustrated in Figure 1 and has been described in detail by Osebe et al [16]. End users access the system via specific apps on smartphones (for patients) and tablets (for providers). Patients use a *patient care wallet* to view their health records, view who accessed records, consent to the sharing of their health data, and communicate with their care providers via text messages. Care providers use a *clinical encounters app* to document care observations and interventions made during encounters in a clinical workflow, view patient records, and initiate patient-consented data sharing processes.

**Table 3:** Key components and capabilities of the proposed digital health wallet platform.

Component	Key capabilities	Targeted recommendation
Care management platform	Mediation of patient-consented health data sharing Blockchain-managed execution of clinical workflows Interoperability across disconnected systems Adheres to FHIR^a^ version 3.0 standard	Supporting interoperability (recommendation 17) Reducing costs of care (recommendation 3)
Patient care wallet	Enables patients to view their health records Enables patients to view who accessed records Enables patients to consent to share health data Support patient-provider outreach conversations	Follow-up and education (recommendation 10) Enhancing patient adherence (recommendation 16)
Clinical encounters app	Enables documentation of care by providers in clinical workflows Enables requests for consent from patients Ensures holistic view of the patient	Adoption of local guidelines (recommendation 18) Enabling self-measurements (recommendation 15)

^a^FHIR: Fast Healthcare Interoperability Resources.

The *application services layer* consists of several components that expose core functionalities to end users. The *application service* submits care observations, personal health data, and outreach conversations generated by end users to an off-ledger database. It also generates metadata that capture events and user actions on medical records. The *care guideline service, patient outreach service, anomalous patterns of care service,* and *transition of care summarization service* perform different functions to provide data viewership, insights, patient summaries, and care recommendations intended to support decision making at the point of care.

The *platform services layer* links the application layer and the blockchain layer to ensure access to blockchain services by other services. This layer consists of a *workflow engine* that orchestrates clinical workflows. Workflows consist of interaction points (encounters) such as triage, consultation, and laboratory investigation. These interaction points can be encoded as chain codes that can be controlled and executed in predefined sequences while specifying the actors, actions, inputs, and outputs pertaining to the workflow. The *Consent Manager* enforces privacy preservation and data protection by ensuring that access and viewership of data in the system is controlled by patients. It empowers patients to give explicit consent to the sharing of their clinical encounters data across organizations. Only authorized users who are onboarded into the system can initiate data sharing processes, and no encounter data can be accessed across separate organizations without valid patient consent.

The *blockchain layer* ensures decentralized trust among participating organizations and patients. It uses smart contracts to map out all encounters in a clinical workflow and ensures that only users with permission can access the encounters and associated data. The layer has an *on-ledger storage* that stores only metadata, encryption keys, and links to data in an off-ledger data store. This ensures the preservation of sensitive patient data that remain stored in off-ledger electronic medical records on-site or ideally in a Health Insurance Portability and Accountability Act (HIPAA)–compliant cloud. The blockchain layer also has an Organizations and Users component for onboarding actors into the system and enforcing access control levels and rules that define users who can create, read, update, or share data in the system.

Such a platform provides value to health care stakeholders in managing not only hypertension but also other diseases, particularly as it takes a patient-centric approach. For patients, the platform would enable easy patient-consented sharing of health data while preserving privacy, better patient engagement, and the potential to monetize personal health records. The availability of clinical and personal health data would all be enabled on mobile devices. For care providers, the system would provide holistic views of patient histories, leading to better diagnoses and treatments. Furthermore, efficient access to patient information would optimize care coordination among care providers. Payers would experience reductions in administrative delays and costs associated with paper records and fragmented systems and a reduction in costs associated with unnecessary or duplicate tests and prescriptions and ease auditability of care provision across health facilities.

## Discussion

### Principal Findings

In this study, we investigated stakeholders’ perspectives on the management of hypertension in the private health sector in Kenya and described the design and functionality of a novel digital health solution for addressing the care continuity and quality challenges in the management of hypertension in the sector. Our findings suggest that the Kenyan private health care sector is a complex, fragmented ecosystem with several challenges such as high cost of care, limited health care literacy, lack of self-management support, and ineffective referral systems. We established that the sector needs health data sharing and decision-making platforms to support care coordination and continuity while acknowledging the fact that addressing all the challenges requires a multifaceted approach.

Interestingly, one of the most significant challenges identified in our study was the lack of sufficient vertical integration between hierarchical tiers of health care delivery. This makes it difficult for practitioners to have a complete understanding of the patient and effectively coordinate efforts to deliver high-quality hypertension care. Appropriate triage and referral from lower-level to higher-level health care institutions is critical to the efficient use of limited resources. Less complex cases should be managed in a community or primary care setting, and increasingly complex cases should be referred to higher-level hospitals. Kenya has articulated this strategy in its health sector referral implementation guidelines [17]. However, the lack of communication and visibility of data collected in clinical encounters across different care facilities makes the referral process unreliable and inefficient. This is true not only for Kenya but also for other countries. For example, in the United States, up to 50% of referrals have no communication between care providers and up to 70% have incomplete documentation [18]. Although there is a dearth of literature on referral practices in low- and middle-income countries, such settings are more likely to experience more difficult referral communication challenges due to higher fragmentation and more scarce resources. Consequently, digital health solutions adopted in these settings should enable patient-consented data sharing while ensuring that care providers have the necessary tools for having a holistic view of longitudinal patient records siloed in separate facilities.

### Comparison With Related Work

Our findings on the health literacy of patients and on the knowledge and skill gaps among care providers mirror the findings of the Healthy Heart study [19]. In the Healthy Heart study [19], only 30% of the interviewed patients knew that heart attacks are associated with high blood pressure, and of those who had heard of hypertension, only 18% identified salt as a risk factor and only 33% identified medication as a method for reducing blood pressure. Furthermore, less than 40% of health care professionals at clinics and dispensaries identified lifestyle changes as methods to address hypertension and less than 75% identified medication as a method to treat hypertension. To improve hypertension health literacy, knowledge, and skills among patients and care providers, policy and organizational approaches such as training and continuous medical education are required. In addition, digital health solutions, such as the one proposed in this study, should have capabilities that support guideline-based decision-making and patient engagement and self-management.

Overall, Kenya can learn from its significant experience in tackling the HIV epidemic. The key themes identified in this study have been addressed, to some extent, in the ongoing management of HIV in Kenya. For example, the study by Oduor et al [20] suggests that the use of technology to manage HIV is influenced by the roles and routines of the patients and clinicians, and such use can inform the design of technologies that can support patients living with comorbid HIV and hypertension. However, directly importing the HIV model for additional conditions would be a challenge, both due to the overall cost of the model (HIV accounts for over 35% of total health expenditure in Kenya) and funding sources (over 70% of HIV care is funded by international organizations). Solutions must be identified for hypertension and other NCDs that learn from the experiences of HIV management and seek more cost-effective mechanisms of managing these conditions. The solutions should be patient-centered and seek to improve health outcomes, as exemplified in the widely adopted chronic care model [21]. Solution frameworks such as the Digital Health Wallet Platform proposed in this study present valuable approaches for enabling care continuity and quality within fragmented health care systems. Such solutions can be used to improve the efficiency of care coordination and patient referrals by supporting communication between care providers, eliminating unnecessary paperwork, and reducing duplicate services. Such solutions are useful for promoting patient engagement; enhancing medication adherence, safety, and effectiveness; and empowering patients with the management of their health data.

The usability and feasibility of digital health solutions for hypertension and other NCDs should be adequately investigated while considering the important barriers such as low digital literacy among users, high costs of implementation and maintenance, unreliable infrastructure, and weak regulatory frameworks. On the basis of the findings of this study, we have developed the Digital Health Wallet Platform [16] and are in the process of testing its feasibility for the clinical management of HIV and comorbid hypertension and/or diabetes across 3 clinical workflows. We have also identified new research directions that are complementary to the platform, which are important for improving health care delivery in settings such as Kenya. These areas, which are critical for supporting clinical encounter analytics, include context-based abstractive text summarization [22] and the detection of anomalous patterns of care [23,24] and are actively being pursued by our team.

### Conclusions

This study identified key challenges faced by care providers managing hypertension in the Kenyan private health care sector. Our findings suggest that significant policy, organizational, and infrastructural changes are required to address these challenges and ensure care quality and continuity. Multiple partners, including health care payers, health care providers, health care regulators, and technology partners, are required to come together in this endeavor. Additional studies should investigate how digital health data interoperability can be achieved within fragmented ecosystems such as the private sector in health care in Kenya by enabling patients to control and manage the sharing of their data while ensuring that care providers have a holistic view of the patient during any encounter.

## Abbreviations

CPD: continued professional development
FHIR: Fast Healthcare Interoperability Resources
NCD: noncommunicable disease
